# T_1-2_N_1_M_0_ nasopharyngeal carcinoma chemotherapy or not: A retrospective study

**DOI:** 10.1371/journal.pone.0279252

**Published:** 2023-03-02

**Authors:** Pei-Jing Li, Ming Chen, Ye Tian

**Affiliations:** 1 Department of Radiotherapy & Oncology, The Second Affiliated Hospital of Soochow University, Institute of Radiotherapy & Oncology, Soochow University, Suzhou Key Laboratory for Radiation Oncology, Suzhou, China; 2 Department of Radiation Oncology, Cancer Hospital of the University of Chinese Academy of Sciences (Zhejiang Cancer Hospital), Institute of Cancer and Basic Medicine (IBMC), Chinese Academy of Sciences, Hangzhou, China; 3 Department of Radiation Oncology, Sun Yat-sen University Cancer Center, State Key Laboratory of Oncology in South China, Collaborative Innovation Center for Cancer Medicine, Guangzhou, China; Gachon University Gil Medical Center, REPUBLIC OF KOREA

## Abstract

**Background:**

Radiotherapy (RT) combined with chemotherapy is the standard treatment for T_1-2_N_1_M_0_ nasopharyngeal carcinoma (NPC) based on conventional radiotherapy. However, intensity-modulated radiotherapy (IMRT) has narrowed the treatment gap between RT and chemoradiotherapy. Thus, this retrospective study aimed to compare the efficacy of RT and chemoradiotherapy (RT-chemo) in treating T_1-2_N_1_M_0_ NPC in the IMRT era.

**Materials and methods:**

From January 2008 to December 2016, 343 consecutive patients with T_1-2_N_1_M_0_ NPC in two cancer centers were included. All patients received RT or RT-chemo, chemotherapy including induction chemotherapy (IC) + concurrent chemoradiotherapy (CCRT), CCRT, or CCRT + adjuvant chemotherapy (AC). The number of patients who received RT, CCRT, IC + CCRT, and CCRT + AC was 114, 101, 89, and 39. The survival rates were analyzed using the Kaplan-Meier method and compared using the log-rank test. Multivariable analysis was performed to identify valuable prognostic factors.

**Results:**

The median follow-up time for survivors was 93 (range: 55–144) months. The 5-year overall survival (OS), progression-free survival (PFS), locoregional failure-free survival (LRFFS), and distant metastasis-free survival (DMFS) for the RT-chemo and RT groups were 93.7%, 88.5%, 93.8%, 93.8% and 93.0%, 87.7%, 91.9%, 91.2%, respectively (P>0.05 for all outcomes). No significant survival differences were found between the two groups. The T_1_N_1_M_0_ or T_2_N_1_M_0_ subgroup analysis showed that treatment outcomes had no significant differences between the RT and RT-chemo groups. After adjusting for various factors, treatment mode was not identified as an independent prognostic factor for all survival rates.

**Conclusions:**

In this study, outcomes of T_1-2_N_1_M_0_ NPC patients treated by IMRT alone were comparable to chemoradiotherapy, supporting the omission or postponement of chemotherapy.

## Introduction

Nasopharyngeal carcinoma (NPC) has an extremely skewed geographic distribution. Over 70% of the cases were reported in East and Southeast Asia [1, 2]. Induction chemotherapy (IC) followed by concurrent chemoradiotherapy (CCRT) or CCRT ± adjuvant chemotherapy (AC) is currently the standard treatment for patients with stage II NPC based on two-dimensional conventional radiotherapy (2DRT) [3]. Intensity-modulated radiotherapy (IMRT) has been widely used since1990s. The 5-year overall survival (OS) rate for stage II NPC patients increased from 85% using 2DRT to 95% using IMRT alone [4–6]. With the excellent outcomes of IMRT, chemotherapy is becoming unnecessary for patients with T_2_N_0_M_0_ NPC unless they have high-risk features such as bulky tumor volumes or high plasma/serum EBV DNA copy numbers [7]. However, N-positive disease is an established high-risk factor for distant metastasis, making patients with T_1-2_N_1_M_0_ NPC a unique subgroup within the stage II disease [8]. There are no definitive evidence-based recommendations for managing T_1-2_N_1_ disease in the IMRT era [7]. Only one retrospective study with small sample size and short follow-up focusing on T_1-2_N_1_M_0_ NPC (AJCC 6th edition) patients explored the efficacy of CCRT [9] in the context of IMRT. Therefore, this study aimed to explore the role of chemotherapy in T_1-2_N_1_M_0_ NPC patients treated with radical IMRT in a large cohort and with extended follow-up.

## Materials and methods

### Patients

This study retrospectively collected data of 343 consecutive patients with T_1-2_N_1_M_0_ NPC from two cancer centers between January 2008 and December 2016. The inclusion criteria were histopathologically confirmed non-keratinizing NPC, T_1-2_N_1_M_0_ disease were re-staged by the American Joint Committee on Cancer (AJCC) TNM 8th edition staging system, age 18–80 years, completion of radical radiation, and without other malignancies. Demographic (age, gender, etc.) and clinical (stage, treatment, disease outcomes and survival, etc.) data were collected. The study was conducted in accordance with the Declaration of Helsinki for all human or animal experimental investigations and approved by the Ethics Committee of Zhejiang Cancer Hospital(IRB-2021-90). The informed consent requirement has been waived due to the retrospective nature of this study.

### Treatment and follow-up

Whether patients received RT alone or chemoradiotherapy depended on a combination of the individual’s will and the physician’s recommendation after case discussion because the treatment of patients with stage II NPC was controversial during the time patients were included in this study. The induction regimens were based on platinum: PF (cisplatin/nedaplatin + 5-fluorouracil), TP (docetaxel + cisplatin), or TPF (docetaxel, cisplatin + 5-fluorouracil), repeated every 21 days for 2–3 cycles. Concurrent chemotherapy regimens were cisplatin or nedaplatin, administered weekly (3–6 cycles) or every 3 weeks (2–3 cycles). Adjuvant chemotherapy regimens were PF, TP, or capecitabine, administered every 3 to 4 weeks for 2–4 cycles. All patients underwent radical IMRT. Simultaneously modulated accelerated radiation therapy (SMART) technique was used. We delivered 2.12–2.18 Gy/F to PGTV (planning target for the primary tumor and positive lymph node) while delivering 2 Gy/F to PTV1 (planning target for the high-risk subclinical lesions) and 1.8 Gy/F to PTV2 (planning target for the low-risk subclinical lesions). The total dose for PGTV, PTV1, and PTV2 was 68–70 Gy, 60–64 Gy, and 54–58 Gy, completed in 30-32F, 5F per week. The detailed IMRT plan was the same as previous studies [10, 11]. Outpatient workup is the main form of follow-up. The final follow-up date was August 31, 2021.

### Statistical analysis

All patients were divided into the RT and chemoradiotherapy groups (RT-chemo, including IC+CCRT, CCRT, CCRT+AC). We evaluated treatment outcomes of locoregional failure-free survival (LRFFS), distant metastasis-free survival (DMFS), progression-free survival (PFS), and OS in this study. Progression is defined as disease progression or death from any cause. Cancer-specific survival (CSS) was added to highlight cancer-related events and defined as individuals who die of NPC. All endpoints were defined as the time from the onset of treatment to the time of event occurrence or the last follow-up in the absence of an event. The Kaplan–Meier method was used to analyze the time-to-event endpoints. The log-rank test was performed to compare differences between groups. Hazard ratios (HRs) and 95% confidence intervals (CIs) were calculated using Cox regression analysis. Multivariate analyses were used to identify valuable predictive factors. Categorical and continuous variables were compared using Pearson’s χ^2^ test, Fisher’s exact test, and t-test. R software (R version 4.0.2) and SPSS version 25.0 (IBM Corp., Armonk, NY, USA) were used for data analyses. Statistical significance was set at p < 0.05.

## Results

### Patient characteristics

The median follow-up time for the 303 survivors was 93 (range: 55–144) months. A total of 284 (93.7%) survivors were followed up for at least 5 years. The median survival was not reached. The male-to-female ratio was 2.61:1. The baseline characteristics were well-balanced between the groups in the whole group analysis and the T_1_/T_2_N_1_M_0_ subgroup analysis (all P > 0.05). The details are summarized in Table 1 and S1 Table.

**Table 1:** Baseline characteristics.

Characteristics	RT group	RT-chemo group	P-value
	n = 114	n = 229	
**Age (years/median)**	47 (20–79)	48 (21–77)	0.112
**Sex**			0.510
**Male**	85 (74.6%)	163 (71.2%)	
**Female**	29 (25.4%)	66 (28.8%)	
**Histology, WHO type**			0.826
**NKDC**	3 (2.6%)	7(3.1%)	
**NKUC**	111 (97.4%)	222 (96.9%)	
**AJCC 8th edition stage**			0.699
**T**_**1**_**N**_**1**_**M**_**0**_	34 (29.8%)	73 (31.9%)	
**T**_**2**_**N**_**1**_**M**_**0**_	80 (70.2%)	156 (68.1%)	
**RTT(days/mean)**	44.5 (41–51)	44.9 (41–51)	0.113

Data are presented as n (%) or mean/median (range). *P value*s are calculated using the χ^2^ test or t-test. RT = radiotherapy, RT-chemo = chemoradiotherapy, NKDC = non-keratinizing differentiated carcinoma, NKUC = non-keratinizing undifferentiated carcinoma, RTT = radiation treatment time.

### Failure patterns

Overall, 39 deaths were recorded up to the last follow-up: 15 (13.2%) in the RT group and 24 (10.5%) in the RT-chemo group without statistical significance between the groups (P = 0.462). Thirty-six patients died due to cancer-related causes, and no difference was found between the two groups either (12.3% vs. 9.6%, P = 0.447). A total of 52 patients experienced treatment failure. The progression, locoregional relapse, and distant metastasis had no significant differences between the two groups (all P > 0.05, Table 2). In subgroup analyses, death, cancer-specific death, progression, relapse, and metastasis had no significant differences between the RT alone and RT-chemo groups either in the T_1_N_1_ or T_2_N_1_ populations (all P > 0.05, Table 2). We compared the event incidence between the CCRT, IC + CCRT, CCRT + AC, and RT alone populations regarding different chemotherapy sequences. Similarly, no significant differences were found in any analyzed events (S2 Table).

**Table 2:** Comparison of events incidence between the RT and RT-chemo groups.

Events	RT group	RT-chemo group	OR (95% CI)	P value
			RT vs. RT + chemo	
**Whole group**	n = 114	n = 229		
**Death due to any reason**	15 (13.2%)	24 (10.5%)	0.773 (0.388–1.538)	0.462
**Death due to NPC**	14 (12.3%)	22 (9.6%)	0.759 (0.373–1.546)	0.447
**Progression**	20 (17.5%)	32 (14.0%)	0.763 (0.415–1.406)	0.385
**Relapse**	13 (11.4%)	18 (7.9%)	0.663 (0.313–1.406)	0.281
**Metastasis**	11 (9.6%)	17 (7.4%)	0.751 (0.339–1.661)	0.478
**T**_**1**_**N**_**1**_**M**_**0**_ **subgroup**	n = 34	n = 73		
**Death due to any reason**	3 (8.8%)	8 (11.0%)	1.272 (0.315–5.127)	0.735
**Death due to NPC**	3 (8.8%)	7 (9.6%)	1.096 (0.265–4.526)	0.899
**Progression**	5 (14.7%)	8 (11.0%)	0.714 (0.215–2.370)	0.581
**Relapse**	4 (11.8%)	5 (6.8%)	0.551 (0.138–2.199)	0.394
**Metastasis**	3 (8.8%)	4 (5.5%)	0.599 (0.126–2.839)	0.515
**T**_**2**_**N**_**1**_**M**_**0**_ **subgroup**	n = 80	n = 156		
**Death due to any reason**	12 (15.0%)	16 (10.3%)	0.648 (0.290–1.445)	0.286
**Death due to NPC**	11 (13.8%)	15 (9.6%)	0.667 (0.291–1.530)	0.337
**Progression**	15 (18.8%)	24 (15.4%)	0.788 (0.387–1.603)	0.510
**Relapse**	9 (11.3%)	13 (8.3%)	0.717 (0.293–1.757)	0.466
**Metastasis**	8 (10.0%)	13 (8.3%)	0.818 (0.324–2.064)	0.670

Values are shown as n (%). ORs and *P value*s are calculated using the χ^2^ test. NPC = nasopharyngeal carcinoma, RT = radiotherapy, RT-chemo = chemoradiotherapy, OR = odds ratio, CI = confidence interval.

### Survival outcomes

**Fig 1:**
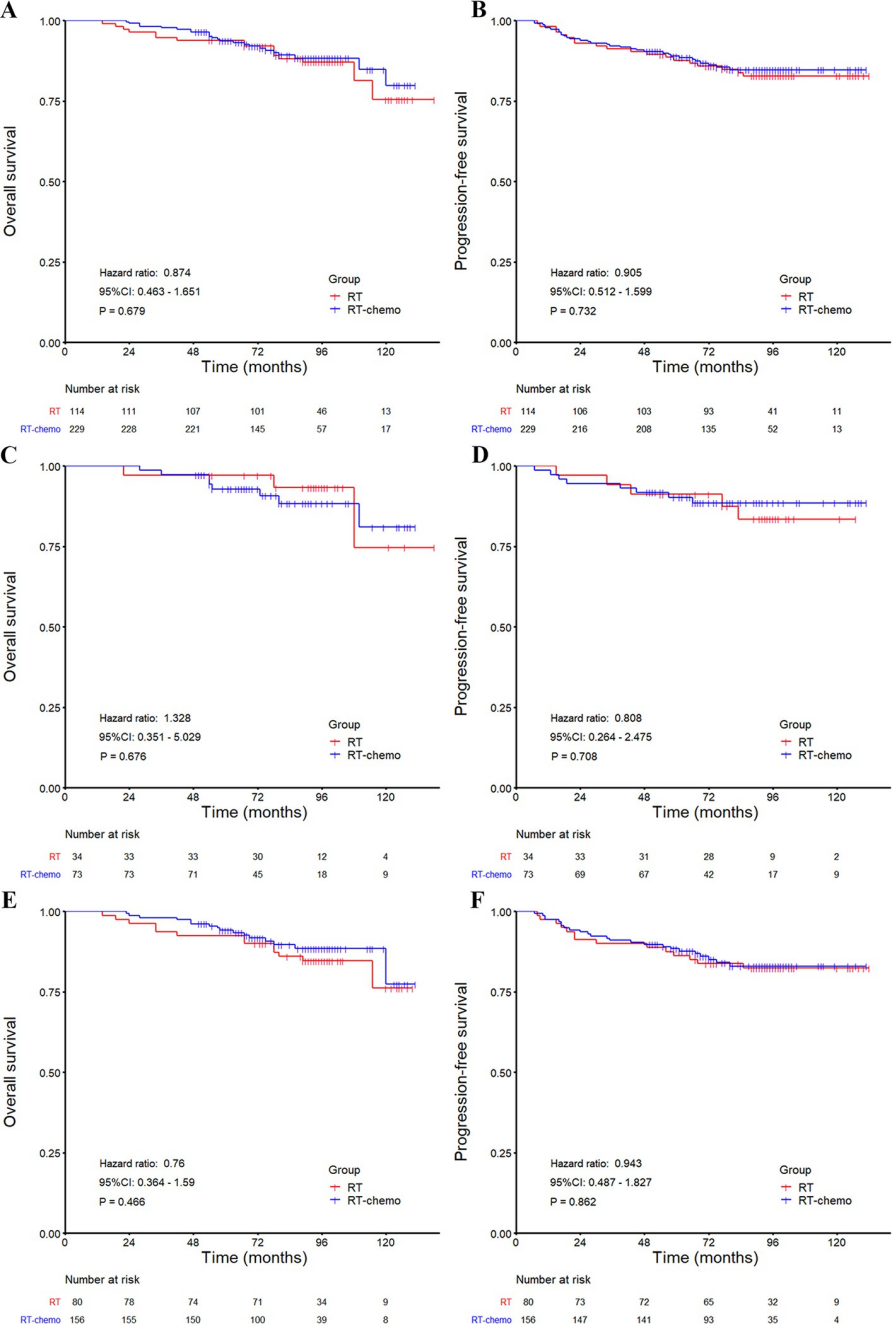
Kaplan–Meier survival curves for the RT and RT-chemo groups. (A–B) whole group analysis: overall survival and progression-free survival, (C–D) T_1_N_1_ subgroup analysis: overall survival and progression-free survival, (E–F) T_2_N_1_ subgroup analysis: overall survival and progression-free survival. HRs are calculated with an unadjusted Cox proportional hazards model. P values are calculated with the unadjusted log-rank test. CI = confidence interval, RT = radiotherapy, RT-chemo = chemoradiotherapy.

**Fig 2:**
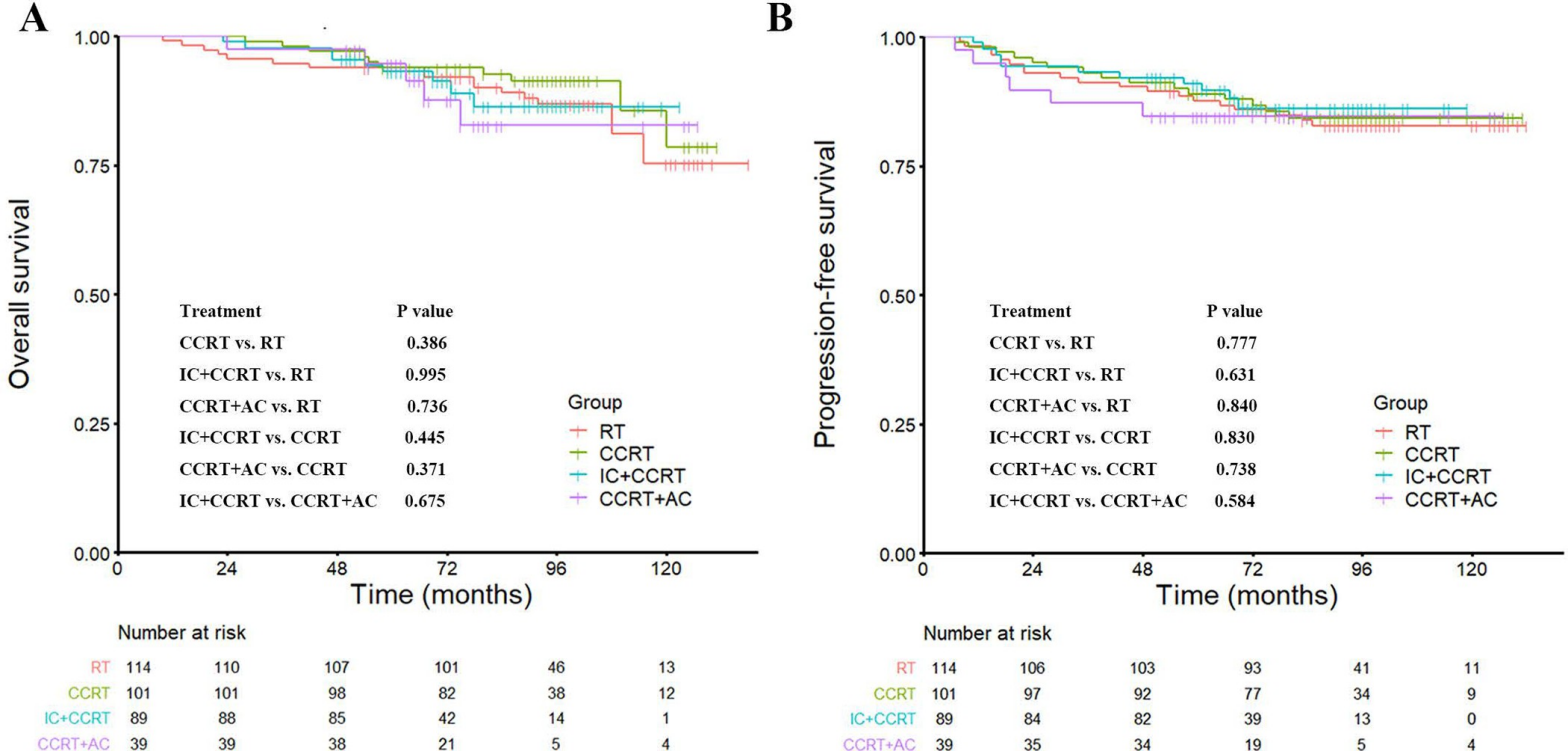
Kaplan–Meier survival curves for the RT, CCRT, IC + CCRT, and CCRT + AC groups. (A) overall survival, (B) progression-free survival. P values are calculated with the χ^2^ test. Abbreviation: RT = radiotherapy, CCRT = concurrent chemoradiotherapy, IC = induction chemotherapy, AC = adjuvant chemotherapy.

The 5- and 10-year OS rates of the RT-chemo group were similar to those of the RT group (93.7% vs. 93.0%, and 79.8% vs. 76.2%, HR: 0.874, 95%CI: 0.463–1.651, P = 0.679, Fig 1A and S3 Table). The RT-chemo group did not show significant improvement in the 5- and 10-year PFS, CSS, LRFFS, or DMFS rates compared with the RT group (Fig 1B, S1A–S1C Fig, and S3 Table). Similar trends were observed in the T_1_N_1_ and T_2_N_1_ subgroup analyses. There was no significant survival difference between the RT and RT-chemo groups, either in T_1_N_1_M_0_ or T_2_N_1_M_0_ disease population (all P > 0.05, Fig 1C–1F, and S1D–S1I Fig). When different chemotherapy sequences were analyzed separately as the RT, CCRT, IC + CCRT, and CCRT + AC groups, no significant survival differences were found among the groups (all P > 0.05, Fig 2, S2 Fig).

### Multivariate analysis

**Fig 3:**
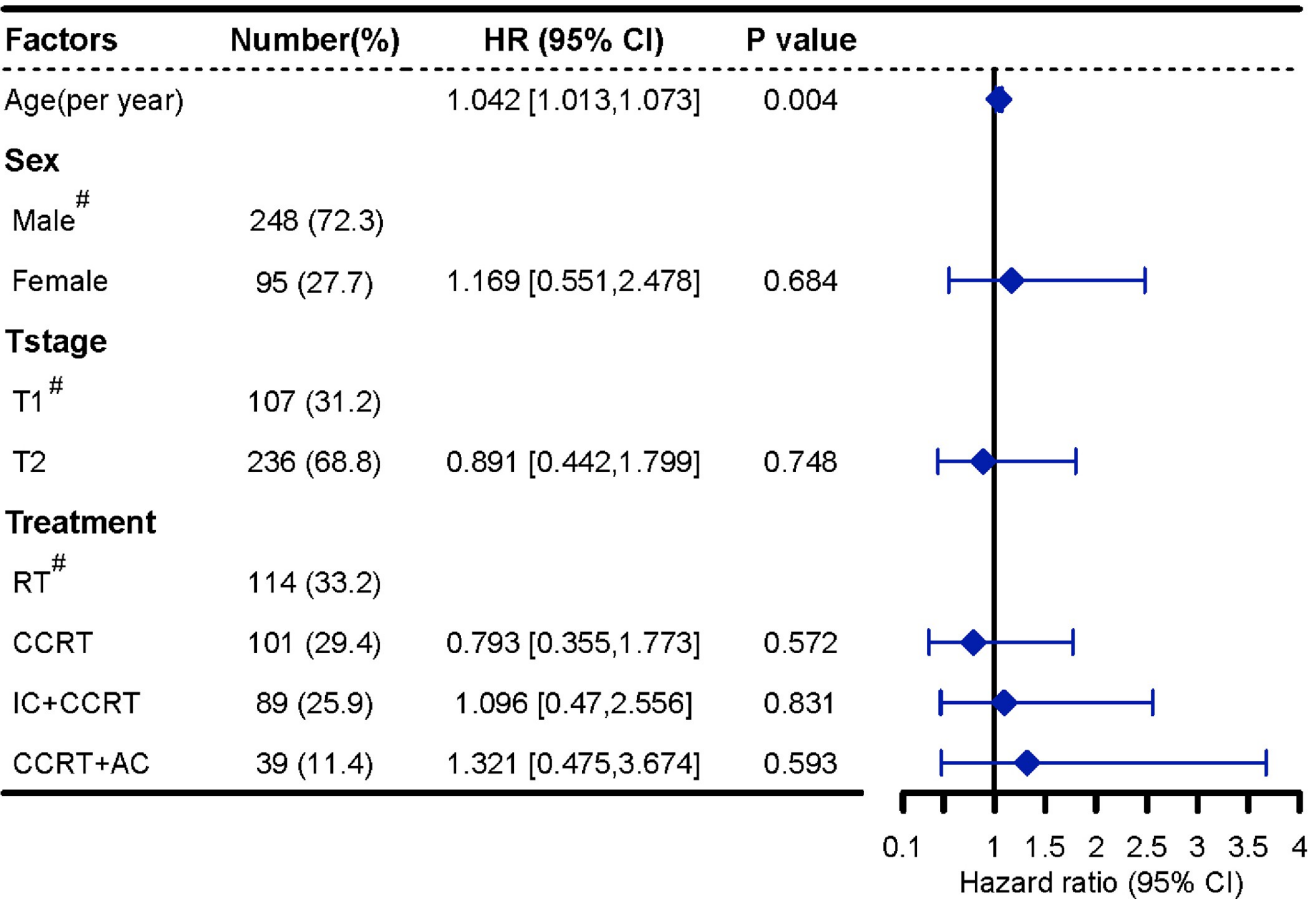
Cox Forest plots for the death of patients with T_1-2_N_1_M_0_ NPC. HRs and P values are calculated with the adjusted Cox proportional hazards model. HR = hazard ratio, CI = confidence interval, RT = radiotherapy, CCRT = concurrent chemoradiotherapy, IC = induction chemotherapy, AC = adjuvant chemotherapy. ^#^ = reference category.

In multivariable analyses, after adjusting for age, sex, and T stage, the combined chemotherapy did not significantly improve OS, PFS, CSS, LRFFS, and DMFS. Treatment mode was not identified as an independent prognostic factor for all survival rates (all P >0.05, Fig 3 and S4 Table). Then, age was an independent prognostic factor for OS, CSS, PFS, and DMFS and inversely related to these outcomes. However, age was not a predictor of LRFFS.

## Discussion

The present retrospective study demonstrated that IMRT alone could achieve an equivalent prognosis to chemoradiotherapy for treating patients with T_1-2_N_1_M_0_ NPC in the IMRT era.

Cheng et al. [12] retrospectively reviewed 44 patients with I-II NPC (AJCC 5th edition): 11 with stage I disease and 1 with stage II NPC received RT alone, and 32 patients with stage II NPC were treated with CCRT. The 3-year LRFFS and DMFS rates were similar in the two arms suggesting that the unfavorable prognosis of stage II NPC patients was reversed by concurrent chemotherapy. A post-hoc analysis conducted by Chua et al. [13] indicated that adding IC to RT (n = 98) significantly improved OS (79% vs. 67%, P = 0.048) and DMFS (86% vs. 71%, P = 0.005) of stage II NPC patients (AJCC 5th edition) comparing with RT alone (n = 110). Chen and Li [4, 14] carried out a randomized clinical trial revealing that CCRT was significantly superior to 2DRT in treating stage II NPC (Chinese 1992 stage) (OS: 83.6% vs. 65.8%, CSS: 86.2% vs. 71.9%, PFS: 76.7% vs. 64.0%, DMFS: 94.0% vs. 83.3%, P < 0.05 for all). A retrospective study focused on T_2_N_1_M_0_ patients demonstrated that cisplatin-based CCRT significantly improved relapse-free survival (91.5% vs. 77.3%, P = 0.008) but not overall survival when comparing RT alone [15]. Studies confirming the therapeutic advantage of chemoradiotherapy have focused more on the treatment of stage II NPC patients other than the T_1-2_N_1_M_0_ subgroup. In addition, all the above studies were based on the 2DRT technique.

IMRT generates a uniform dose distribution in the target region and a sharp dose gradient transition to the adjacent vital organs, allowing better protection of the normal tissue and appropriately increased dose of the target volume. Then, the SMART technique shortens the time of radiotherapy. Lai et al. [16] retrospectively reviewed the outcomes of 1276 NPC patients who received 2DRT or IMRT, showing that IMRT provided survival benefits, especially in early-stage T disease. In the IMRT era, the efficacy gap between RT alone and chemoradiotherapy has narrowed. Whether the ceiling effect appears in early-stage NPC treatment using IMRT is worth further discussion. The treatment outcomes of adding chemotherapy to RT in the different eras were utterly different. One study revealed that CCRT ± AC had no advantages in improving the prognosis of patients with II NPC (AJCC 7th edition) compared with IMRT alone [5]. Su et al. [6] retrospectively reviewed 249 patients with stage II NPC (AJCC 7th edition) and indicated that there was no significant difference in prognosis between IMRT alone (n = 106) and CCRT (n = 143). Chen et al. [17] conducted a prospective study in stage II NPC patients (AJCC 7th edition) and revealed that CCRT + AC(n = 81) did not gain favorable 5-year OS and DMFS rates when compared with IMRT (n = 79) (OS: 91.4 vs. 88.6%, DMFS: 93.82% vs. 93.67%, all P > 0.05). Moreover, a phase II clinical study [18] of 84 patients with stage II NPC (AJCC 7th edition) explored the efficacy of concurrent chemotherapy. The OS, local failure-free survival (LFFS), regional failure-free survival (RFFS), disease-free survival (DFS), and DMFS of the IMRT (n = 43) and CCRT (n = 41) groups were 100% vs. 94.0%, 93.0% vs. 89.3%, 97.7% vs. 95.1%, 90.4% vs. 86.6%, and 95.2% vs. 94.5%, respectively (P > 0.05 for all). However, there were different voices. Sun et al. [19] retrospectively reviewed 3808 stage II patients treated from 1990 to 2012, indicating that the absence of chemotherapy was still a risk factor for poor prognosis after adjusting for radiotherapy technique (2DRT, 3DRT, and IMRT), family history, personal history, and stage. Studies focused on the T_1-2_N_1_M_0_ disease population are extremely limited. A retrospective study demonstrated that patients with T_1-2_N_1_ NPC (AJCC 5th edition) had a relatively poor prognosis than T_2_N_0_ disease [20]. The authors deemed positive regional lymph node(s) a high-risk factor of distant metastasis and suggested a more aggressive therapy for the T_1-2_N_1_ population. Besides, Luo et al. [8] retrospectively analyzed 69 patients with stage I-II NPC (AJCC 6th edition) from China’s non-endemic region. They found that the T_2_N_1_ classification was a subgroup with a higher risk of distant metastasis. A more apparent separation of the survival curves was observed in the T_2_N_1_ subgroup analyses than in the overall evaluation of the whole group (3-year OS, LRFFS, DMFS were 100% vs. 78.6%, 100% vs. 79.5%, 100% vs. 79.6%, all P < 0.05). By far, only one retrospective study focusing on T_1-2_N_1_M_0_ NPC (AJCC 6th edition) patients explored the efficacy of CCRT [9] in the context of IMRT. A group of paired patients received CCRT (n = 43) or IMRT (n = 43), the 3-year OS, PFS, LRFFS, and DMFS in each group were 100% vs. 94.8%, 89.9% vs. 90%, 92.3% vs. 95%, 97.6% vs. 91.7%, respectively (all P > 0.05). Their results indicated that the omission of chemotherapy did not affect the prognosis. Although with a well-balanced cohort, the sample size was small and the follow-up (median: 37.4 months, range: 4.8–66.2 months) was not long enough in the above study regarding a disease with a relatively good prognosis (5-year OS is more than 90%).

The AJCC NPC staging system has undergone four editions of updates from 1997 to 2017. The T stage of the AJCC 8th edition staging system is markedly different from the previous versions. T_2_ in the latest staging system includes tumors extending to the parapharyngeal space and/or adjacent soft tissue involvement (medial pterygoid, lateral pterygoid, prevertebral muscles), covering a fraction of T_4_ disease in previous editions. Therefore, the results of the above studies must be taken with caution. We re-staged NPC patients in our database according to the AJCC 8th edition staging system and screened 343 patients with T_1-2_N_1_M_0_ NPC for analysis. This retrospective study represents the largest analysis of T_1-2_N_1_M_0_ NPC patients to date, focusing on chemotherapy. Considering that the TNM staging system has long been recognized as the most important prognostic indicator for NPC, we performed a subgroup analysis of T_1_N_1_M_0_ and T_2_N_1_M_0_ diseases. Similar to the overall evaluation, the combination chemotherapy group still did not show any survival advantage in either subgroup. In addition, we divided all patients into the RT, CCRT, IC + CCRT, and CCRT + AC groups. The incidence of events and survival outcomes were not significantly different between the IMRT alone and the other chemoradiotherapy groups. Using IMRT, the radiation dose of the primary tumor and positive retropharyngeal lymph node is almost the same as 2DRT. However, due to the limitation of toxicity, the dose of positive cervical lymph nodes in 2DRT (60–62 Gy) [4] is lower than that of IMRT (66–70 Gy). This difference might explain why IMRT enhances the locoregional control rate and offset the improvement of concurrent chemotherapy on locoregional control. Moreover, advances in imaging technology have made the target volume more accurate in the IMRT era. The present findings corroborate previous reports suggesting that IMRT is sufficient for treating patients with T_1-2_N_1_M_0_ NPC. We believe it may be feasible to omit or postpone chemotherapy in T_1-2_N_1_M_0_ NPC when using IMRT for radiotherapy.

This study also confirms that the failure risk persists 5 years after treatment, which is consistent with the findings of Qin et al. [21] and Chua et al. [20]. Therefore, it is of great importance to maintain a longer follow-up and report long-term results when the data are mature in studies of NPC. The median follow-up of nearly 10 years indicates the reliability of the results in this study. As a retrospective study, this study had some limitations. First, plasma/serum EBV DNA has become an effective prognostic biomarker in recent decades [22, 23], complementing the TNM staging system for selecting patients at a high risk of developing distant metastasis. However, EBV DNA data are lacking in the present study due to incomplete data. Future prospective studies can be designed to better incorporate biomarkers such as plasma/serum EBV DNA levels with the TNM staging system for risk stratification. Second, the toxicity profile of the treatment was not assessed in the present study. Nevertheless, previous studies have shown that chemotherapy increased acute treatment-related toxicities [5, 6, 17, 18]. Overall, our report is noteworthy because of the large population, long-term follow-up, and adoption of multivariate analyses.

## Conclusions

This study showed that T_1-2_N_1_M_0_ NPC patients receiving IMRT and chemoradiotherapy had comparable treatment outcomes, supporting the omission or postponement of chemotherapy. However, prospective studies are warranted to validate the findings in the present study.

## Supporting information

S1 Table Baseline characteristics of the T_1_N_1_M_0_ and T_2_N_1_M_0_ subgroups. (DOCX)

S2 Table Comparison of events incidence in the RT, CCRT, IC + CCRT, and CCRT + AC groups. (DOCX)

S3 Table Survival outcomes of patients in the RT and RT-chemo groups. (DOCX)

S4 Table Multivariable analyses of prognostic factors for all treatment outcomes. (DOCX)

S1 Fig Kaplan–Meier survival curves for the RT and RT-chemo groups. (A–C) Whole group analysis: cancer-specific survival, locoregional failure-free survival, and distant metastasis-free survival; (D–F) T_1_N_1_ subgroup analysis: cancer-specific survival, locoregional failure-free survival, and distant metastasis-free survival; (G–I) T_2_N_1_ subgroup analysis: cancer-specific survival, locoregional failure-free survival, and distant metastasis-free survival. HRs are calculated with the unadjusted Cox proportional hazards model. P values are calculated with the unadjusted log-rank test. CI = confidence interval, RT = radiotherapy, RT-chemo = radiation + chemotherapy.
(TIF)

S2 Fig Kaplan–Meier survival curves for the RT, CCRT, IC + CCRT, and CCRT + AC groups. (A) Cancer-specific survival, (B) Locoregional failure-free survival, (C) Distant metastasis-free survival. P values are calculated with log-rank test (pairwise over strata). RT = radiotherapy, CCRT = concurrent chemoradiotherapy, IC = induction chemotherapy, AC = adjuvant chemotherapy.
(TIF)
