# Supplementary material for: T1-2N1M0 nasopharyngeal carcinoma chemotherapy or not: A retrospective study
Source: PLoS One. 2023 Mar 2;18(3):e0279252. doi: 10.1371/journal.pone.0279252 (PMC9980793; doi:10.1371/journal.pone.0279252)
Supplement: S1 Table — (DOCX) [file pone.0279252.s001.docx]

**S1 Table. Baseline characteristics of the T_1_N_1_M_0_ and T_2_N_1_M_0_ subgroups.**

| **Characteristics** | **RT group** | **RT-chemo group** | **P-value** |
| --- | --- | --- | --- |
| **T_1_N_1_ subgroup** | **n = 34** | **n = 73** |  |
| Age (years/median) | 46 (23–72) | 49 (26–77) | 0.240 |
| Sex |  |  |  |
| Male | 22 (64.7%) | 56 (76.7%) | 0.193 |
| Female | 12 (35.3%) | 17 (23.3%) |  |
| Histology, WHO type |  |  |  |
| NKDC | 1 (2.9%) | 2 (3.0%) | 0.990 |
| NKUC | 33 (97.1%) | 65 (97.0%) |  |
| RTT (days/mean) | 45.0 (42-51) | 45.2 (41-51) | 0.675 |
| **T_2_N_1_ subgroup** | **n = 80** | **n = 156** |  |
| Age (years/median) | 47 (20-79) | 47 (21-72) | 0.253 |
| Sex |  |  |  |
| Male | 63 (78.8%) | 107 (21.3%) | 0.100 |
| Female | 17 (68.6%) | 49 (31.4%) |  |
| Histology, WHO type |  |  |  |
| NKDC | 2 (2.5%) | 5 (3.1%) | 0.798 |
| NKUC | 78 (97.5%) | 157 (96.9%) |  |
| RTT (days/mean) | 44.3 (41-51) | 44.8 (41-51) | 0.127 |
| Data are presented as n (%) or median (range). P values are calculated using the χ² test and t-test. RT = radiotherapy, RT-chemo = chemoradiotherapy, NKDC = non-keratinizing differentiated carcinoma, NKUC = non-keratinizing undifferentiated carcinoma, RTT = radiation treatment time. | | | |
