# Supplementary material for: T1-2N1M0 nasopharyngeal carcinoma chemotherapy or not: A retrospective study
Source: PLoS One. 2023 Mar 2;18(3):e0279252. doi: 10.1371/journal.pone.0279252 (PMC9980793; doi:10.1371/journal.pone.0279252)
Supplement: S2 Table — (DOCX) [file pone.0279252.s002.docx]

**S2 Table. Comparison of events incidence in the RT, CCRT, IC + CCRT, and CCRT + AC groups.**

| **Event** | **RT group n = 114** | **CCRT group n = 101** | **IC + CCRT group n = 89** | **CCRT + AC group n = 39** | **P-value** |
| --- | --- | --- | --- | --- | --- |
| **Death due to any reason** | 15 (13.2%) | 10 (9.9%) | 9 (10.1%) | 5 (12.8%) | 0.850 |
| **Death due to NPC** | 14 (12.3%) | 8 (7.9%) | 9 (10.1%) | 5 (12.8%) | 0.720 |
| **Progression** | 20 (17.5%) | 15 (14.9%) | 11 (12.4%) | 6 (15.4%) | 0.788 |
| **Relapse** | 13 (11.4%) | 9 (8.9%) | 5 (5.6%) | 4 (10.3%) | 0.549 |
| **Metastasis** | 11 (9.6%) | 6 (5.9%) | 8 (9.0%) | 3 (7.7%) | 0.791 |
| Values are shown as n (%). P values are calculated using the χ² test. NPC = nasopharyngeal carcinoma, RT = radiotherapy, CCRT = concurrent chemoradiotherapy, IC = induction chemotherapy, AC = adjuvant chemotherapy. | | | | | |
