# Supplementary material for: T1-2N1M0 nasopharyngeal carcinoma chemotherapy or not: A retrospective study
Source: PLoS One. 2023 Mar 2;18(3):e0279252. doi: 10.1371/journal.pone.0279252 (PMC9980793; doi:10.1371/journal.pone.0279252)
Supplement: S3 Table — (DOCX) [file pone.0279252.s003.docx]

**S3 Table. Survival outcomes of patients in the RT and RT-chemo groups.**

| **Outcomes** | **Whole group** | | **P-value** |
| --- | --- | --- | --- |
|  | **RT-chemo  n = 229** | **RT  n = 114** |  |
| **OS** |  |  | 0.679 |
| **5-year** | 93.7% | 93.0% |  |
| **10-year** | 79.8% | 76.2% |  |
| **CSS** |  |  | 0.773 |
| **5-year** | 93.7% | 93.0% |  |
| **10-year** | 88.4% | 82.1% |  |
| **PFS** |  |  | 0.732 |
| **5-year** | 88.5% | 87.7% |  |
| **10-year** | 84.7% | 82.0% |  |
| **LRFFS** |  |  | 0.456 |
| **5-year** | 93.8% | 91.9% |  |
| **10-year** | 91.1% | 87.9% |  |
| **DMFS** |  |  | 0.537 |
| **5-year** | 93.8% | 91.2% |  |
| **10-year** | 92.0% | 90.3% |  |
| P values are calculated using the unadjusted log-rank test. RT = radiotherapy, RT-chemo = radiotherapy + chemotherapy, LRFFS = locoregional failure-free survival, DMFS = distant metastasis-free survival, PFS = progression-free survival, CSS =cancer-specific survival, OS = overall survival. | | | |
