# Supplementary material for: T1-2N1M0 nasopharyngeal carcinoma chemotherapy or not: A retrospective study
Source: PLoS One. 2023 Mar 2;18(3):e0279252. doi: 10.1371/journal.pone.0279252 (PMC9980793; doi:10.1371/journal.pone.0279252)
Supplement: S4 Table — (DOCX) [file pone.0279252.s004.docx]

**S4 Table. Multivariable analyses of prognostic factors for all treatment outcomes.**

|  | **HR** | **95% CI for HR** | **P value** |
| --- | --- | --- | --- |
| **LRFFS** |  |  |  |
| **Age (per year increase)** | 1.025 | 0.993 - 1.058 | 0.124 |
| **Sex (Female vs. Male)** | 1.946 | 0.745 - 5.085 | 0.174 |
| **T stage (T_2_ vs. T_1_)** | 0.952 | 0.437 - 2.074 | 0.901 |
| **CCRT vs. RT** | 0.827 | 0.353 - 1.937 | 0.661 |
| **IC + CCRT vs. RT** | 0.561 | 0.198 - 1.587 | 0.276 |
| **CCRT + AC vs. RT** | 1.134 | 0.368 - 3.501 | 0.827 |
| **DMFS** |  |  |  |
| **Age (per year increase)** | 1.038 | 1.003 - 1.074 | 0.032 |
| **Sex (Female vs. Male)** | 0.863 | 0.378 - 1.970 | 0.726 |
| **T stage (T_2_ vs. T_1_)** | 0.739 | 0.313 - 1.742 | 0.489 |
| **CCRT vs. RT** | 0.606 | 0.223 - 1.643 | 0.325 |
| **IC + CCRT vs. RT** | 0.979 | 0.392 - 2.443 | 0.963 |
| **CCRT + AC vs. RT** | 0.849 | 0.236 - 3.056 | 0.803 |
| **PFS** |  |  |  |
| **Age (per year increase)** | 1.033 | 1.008 - 1.058 | 0.011 |
| **Sex (Female vs. Male)** | 1.202 | 0.628 - 2.299 | 0.579 |
| **T stage (T_2_ vs. T_1_)** | 0.750 | 0.399 - 1.408 | 0.371 |
| **CCRT vs. RT** | 0.877 | 0.448 - 1.717 | 0.702 |
| **IC + CCRT vs. RT** | 0.800 | 0.381 - 1.678 | 0.554 |
| **CCRT + AC vs. RT** | 1.070 | 0.428 - 2.674 | 0.885 |
| **CSS** |  |  |  |
| **Age (per year increase)** | 1.046 | 1.015 - 1.078 | 0.003 |
| **Sex (Female vs. Male)** | 1.221 | 0.553 - 2.694 | 0.621 |
| **T stage (T_2_ vs. T_1_)** | 0.877 | 0.421 - 1.827 | 0.726 |
| **CCRT vs. RT** | 0.682 | 0.285 - 1.630 | 0.389 |
| **IC + CCRT vs. RT** | 1.091 | 0.465 - 2.559 | 0.842 |
| **CCRT + AC vs. RT** | 1.404 | 0.501 - 3.939 | 0.519 |
| HRs and P values are calculated with an adjusted Cox proportional hazards model. HR = hazard ratio, CI = confidence interval, RT = radiotherapy, CCRT = concurrent chemoradiotherapy, IC = induction chemotherapy, AC = adjuvant chemotherapy, LRFFS = locoregional failure-free survival, DMFS = distant metastasis-free survival, PFS = progression-free survival, CSS = cancer-specific survival. | | | |
