# Supplementary figures and images for: T1-2N1M0 nasopharyngeal carcinoma chemotherapy or not: A retrospective study
Source: PLoS One. 2023 Mar 2;18(3):e0279252. doi: 10.1371/journal.pone.0279252 (PMC9980793; doi:10.1371/journal.pone.0279252)

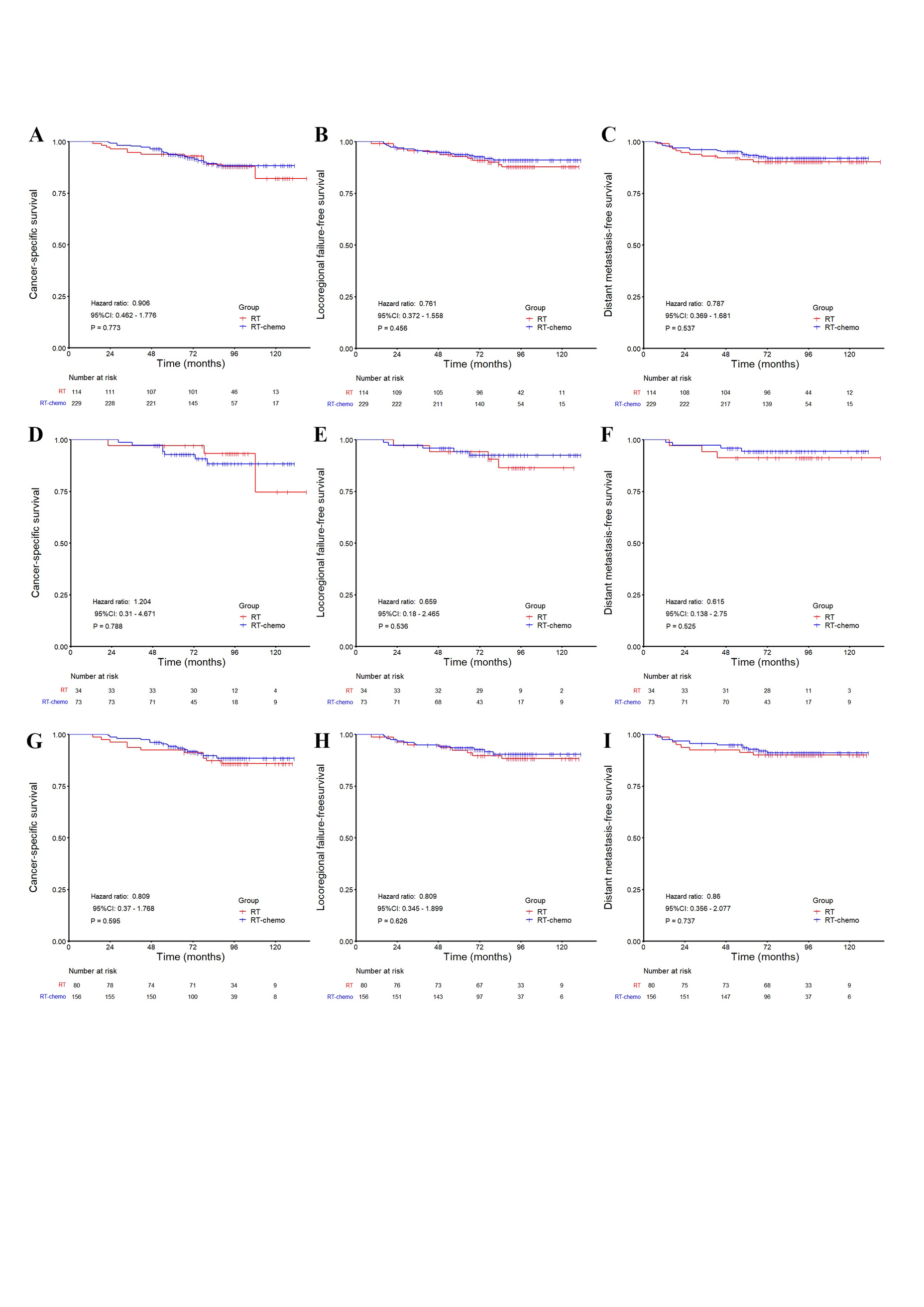

Supplement: S1 Fig — (A–C) Whole group analysis: cancer-specific survival, locoregional failure-free survival, and distant metastasis-free survival; (D–F) T1N1 subgroup analysis: cancer-specific survival, locoregional failure-free survival, and distant metastasis-free survival; (G–I) T2N1 subgroup analysis: cancer-specific survival, locoregional failure-free survival, and distant metastasis-free survival. HRs are calculated with the unadjusted Cox proportional hazards model. P values are calculated with the unadjusted log-rank test. CI = confidence interval, RT = radiotherapy, RT-chemo = radiation + chemotherapy. (TIF) [file pone.0279252.s005.tif]

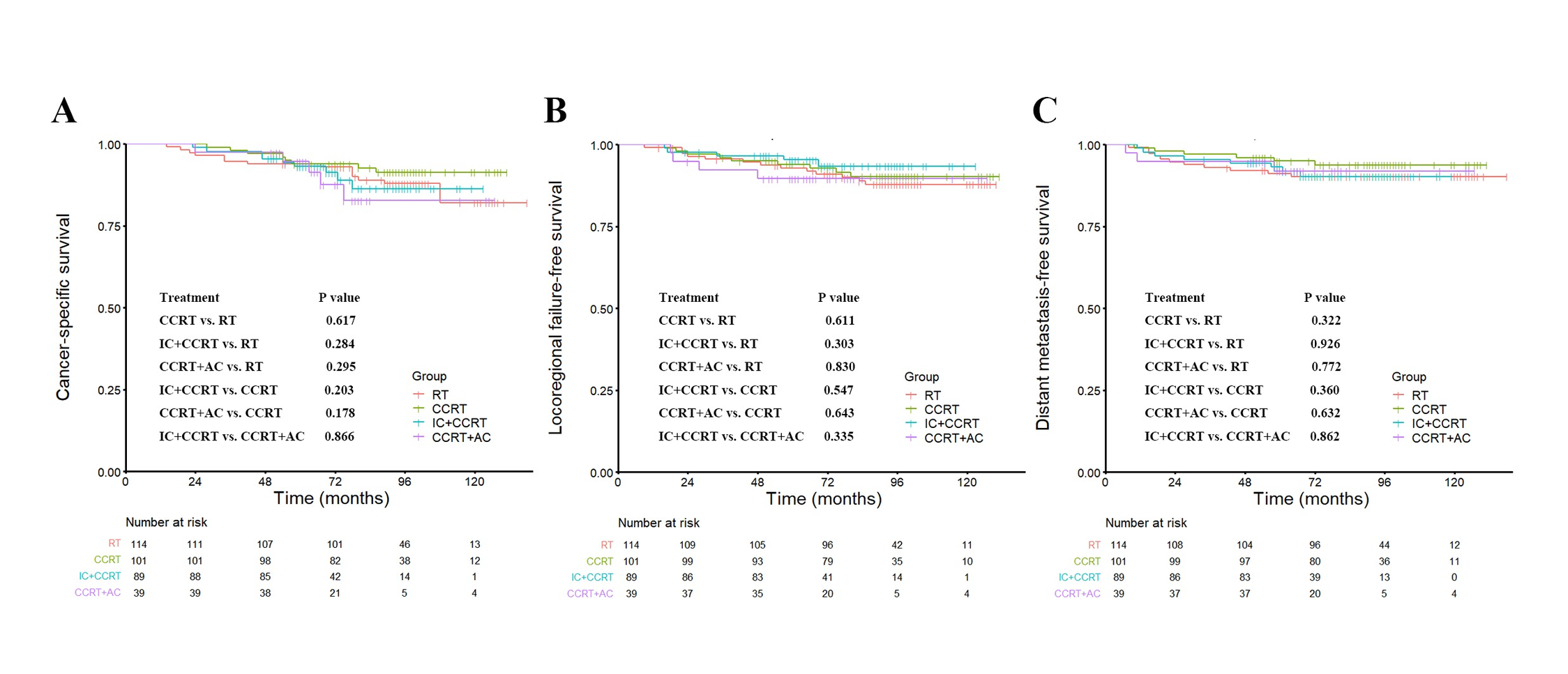

Supplement: S2 Fig — (A) Cancer-specific survival, (B) Locoregional failure-free survival, (C) Distant metastasis-free survival. P values are calculated with log-rank test (pairwise over strata). RT = radiotherapy, CCRT = concurrent chemoradiotherapy, IC = induction chemotherapy, AC = adjuvant chemotherapy. (TIF) [file pone.0279252.s006.tif]
